# Chromatographic Fingerprinting and Food Identity/Quality: Potentials and Challenges

**DOI:** 10.1021/acs.jafc.1c05584

**Published:** 2021-11-23

**Authors:** Luis Cuadros-Rodríguez, Fidel Ortega-Gavilán, Sandra Martín-Torres, Alejandra Arroyo-Cerezo, Ana M. Jiménez-Carvelo

**Affiliations:** Department of Analytical Chemistry, University of Granada, C/Fuentenueva s/n, E-18071 Granada, Spain

**Keywords:** chromatography food authentication, chemometrics and data mining, non-targeted analytical methods

## Abstract

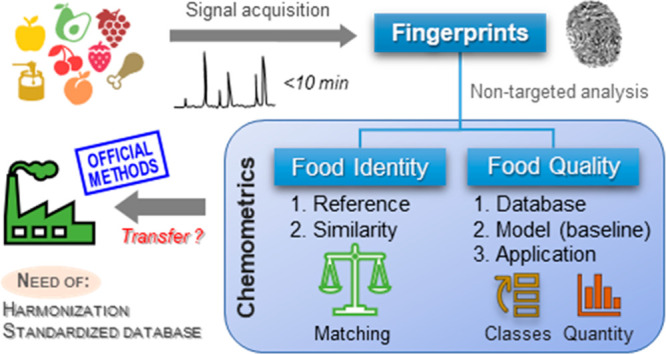

Chromatograms are a valuable source of information about the chemical composition of the food being analyzed. Sometimes, this information is not explicit and appears in a hidden or not obvious way. Thus, the use of chemometric tools and data-mining methods to extract it is required. The fingerprint provided by a chromatogram offers the possibility to perform both identity and quality testing of foodstuffs. This perspective is aimed at providing an updated opinion of chromatographic fingerprinting methodology in the field of food authentication. Furthermore, the limitations, its absence in official analytical methods, and the future directions of this methodology are discussed.

## Introduction

In a broad sense, fingerprinting refers to a recently developed analytical methodology that uses instrumental fingerprints to obtain information about a material feature that is linked to or dependent upon its chemical composition. This feature may refer to the identity of the material concerned, a certain physicochemical property or any other natural property (biological, sensory, etc.), or the presence or amount of one or more chemical compounds in the material. This methodology is mainly associated with the control of food products but is applicable to other fields, such as botany, forensics, cultural heritage, petrochemistry, pharmaceutics, etc.

Instrumental fingerprints refer to signals obtained from a given material using an analytical instrument and subsequently recorded in a suitable storage system. This signal contains implicit information about the chemical composition of the measured material, but this information is hidden and not obviously or explicitly shown nor specific to one or more particular compounds. Therefore, once the fingerprints have been acquired in a first stage, it is mandatory to apply a second stage to extract the useful information using appropriate data-mining methods, being developed under chemometrics, which are particularly designed for this type of chemical data. This is only feasible if a large enough number of representative fingerprints of the studied material is available, so that the mining algorithm is able to locate and read such information embedded in the signal. Analytically valid fingerprints could be obtained applying three strategies:^1^ (i) measuring directly on the original material or the solution resulting from dissolving the whole material, (ii) performing a separation or cleanup step^2^ and measuring on a certain fraction subsequently selected, and (iii) applying a chemical reaction on the original material or the already isolated fraction, i.e., a derivatization reaction, and acquiring the signal on the reaction products derived. The fingerprint resulting from the first strategy is non-specific and mainly reports the composition of the majority compounds. However, there is a chemical pre-selection when applying the second or third strategy, and the available fingerprint is not entirely non-specific, because it is related only to the isolated fraction or the components capable of forming derivatives. Note that the third strategy is consistent with either of the two previous strategies.

It is precisely the no specificity that characterizes an instrumental fingerprint and makes it different from other types of signals or data sets. In our opinion, the term analytical fingerprint should not be used to denote the outcome of collecting data obtained from different sources or analytical methods. However, this is not a shared opinion, and there are authors who consider fingerprints the compilation of multiple analytical parameters^3^ or even a data set associated with representative molecular markers.^4,5^ In these cases, the term “analytical profile” better describes the issue.^6^

**Figure 1:**
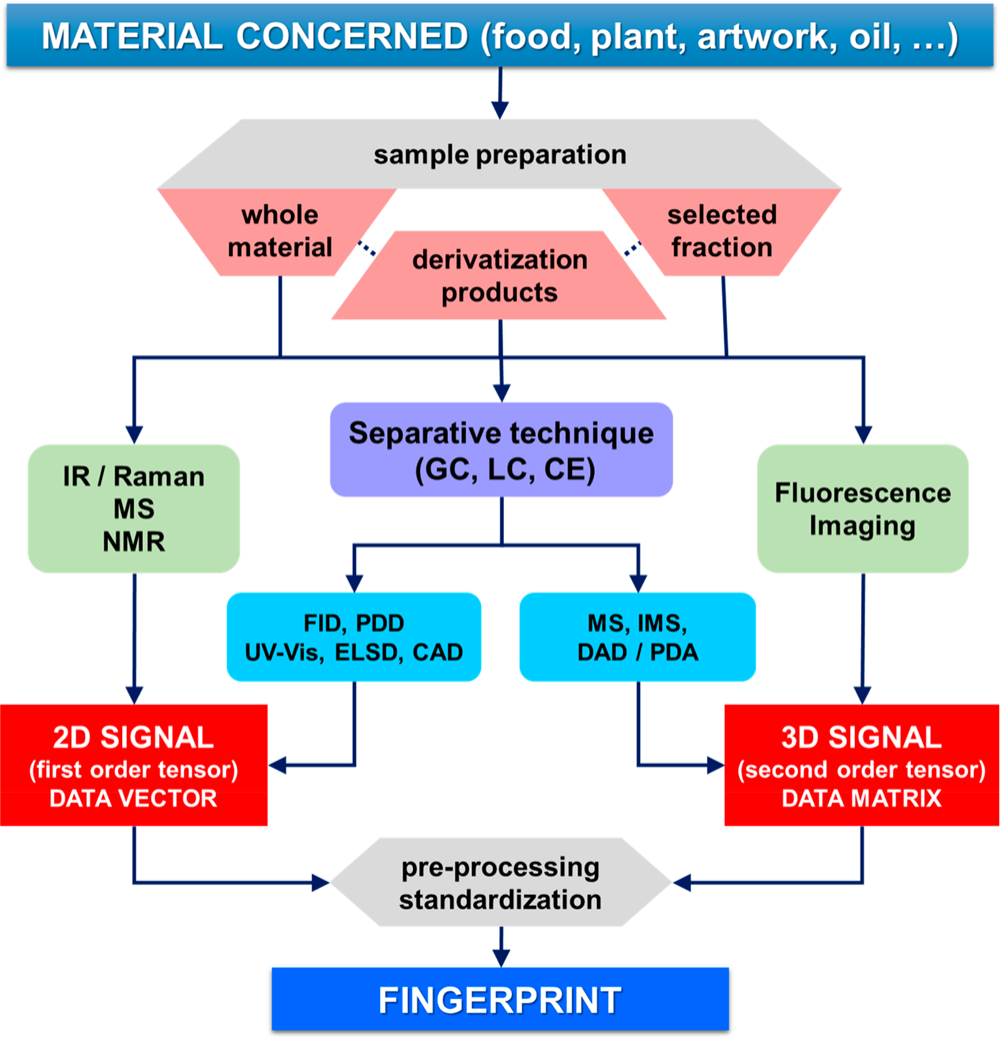
Types of analytical fingerprints.

A fingerprint is generally linked to a two-dimensional (2D) signal taking the shape of a curve (absorption spectrum, voltammogram or any other electrochemical curve, thermogram, kinetic curve, chromatogram, etc.) or a three-dimensional (3D) signal defined by a surface or an image (fluorescence spectrum, comprehensive 2D chromatogram, spectrum–chromatogram, optical or thermal image, map, etc.), although higher dimensionality signals are also feasible. A number of analytical techniques are able to provide fingerprints,^2,7^ e.g., molecular spectroscopy, including imaging (optical, thermal, acoustic, etc.), nuclear magnetic resonance (NMR), mass spectrometry and ionic mobility spectrometry (MS and IMS), voltamperometry, or e-sensing. The case of the separative techniques is particular in that they do not generate signals by themselves but rather the signals are obtained by the measuring device (or detector) coupled to the end of the separative stage. This separative stage adds an additional time dimension to those of the measuring device. Dependent upon the type of detector, 2D fingerprints (e.g., conventional chromatograms or electropherograms) or higher dimensionality fingerprints (e.g., spectrum–chromatograms or images) could be obtained. In all cases, these signals may be referred to as chromatographic or electrophoretic fingerprints. Figure 1 graphically summarizes and describes the different types of fingerprints.

The chemical information implicit in the fingerprint depends upon the basis of the analytical technique used. Therefore, before applying a particular fingerprint, the most suitable analytical technique should be selected to ensure that the information on interest can be embedded in the measured signal. It should not be forgotten that data-mining methods do not perform miracles and cannot extract information that is not already contained in the acquired signal. As examples, a near-infrared (NIR) fingerprint depends upon the particular chemical bonds in each functional molecular groups; a ^1^H NMR fingerprint is linked to the different molecular environments of bonded hydrogens; or a MS fingerprint is associated with the mass profile of the molecular fragments arising from all molecules. The coupling of a separative technique makes the fingerprint information easier to relate to the molecular composition. However, it should be noted that a well-resolved chromatogram represents a profile of compounds and never constitutes a fingerprint because each peak of the signal specifically provides information on a single component (graphically, it resembles a bar chart) and the non-specificity trait is lost.

Therefore, analytical fingerprinting could be defined as an analytical methodology aimed at obtaining specific information linked to the chemical composition of a given material about its identity or about a qualitative or quantitative distinctive quality from a non-specific instrumental signal (fingerprint) that contains the information on interest in a non-obvious and non-explicit way and needs to be extracted by applying specific data-mining methods (chemometrics). Note that this meaning differs from the usual meaning used in certain omics sciences. This issue will be discussed later in the next section.

Under the umbrella of this operational definition, the aim of this perspective is to provide an updated overview of the current state of fingerprinting methodology in the field of food quality and authenticity, with special emphasis on its potential as an analytical tool capable of establishing and assuring the identity and quality of food products. Finally, a section will be devoted to describe future perspectives, focusing on two key challenges: the harmonization of this methodology and the generation of instrument-agnostic chromatographic fingerprint databases that could be universally used because they are independent of the instrument used.

## Food Quality and Food Authentication Focus

The development and effective application of chromatographic fingerprinting involves the sequential achievement of a number of steps, so that each step cannot be started until the previous step has been finished. The process begins by selecting the most appropriate analytical procedure and acquiring and subsequent filing of the raw analytical signals, using any of the strategies discussed in the Introduction. This selection involves a triple decision: (i) the sample preparation method, (ii) the chromatographic mode and conditions, and (iii) the signal acquisition settings in the measuring device.

The raw chromatograms should then be pre-processed and collected to generate a database. From this stage on, fingerprints are available because they always have to be referred to the pre-processed signals. Signal pre-processing is critical and should be decided carefully because the final outcome may be different depending upon how it is performed. In addition, the pre-processing needs to be clearly defined, so that it can be applied under the same conditions when it is required to increase the database with new signals.

From this point on, data-mining tools may be applied. There is a wide battery of data-handling methods that may be selected, and the use of one or the other will depend upon the type of information required. In this regard, it would be appropriate to remember the sequence of tiers of the analytical information: detection, identification, typing, quantitation, and distribution. The different tiers could be applied to different analytical targets, i.e., to individual chemical components (analytes), to empirically defined chemical parameters or indices, or to materials considered as a whole. The meaning of each of these terms is sufficiently well-known, and a description is beyond the scope of this perspective, but the reader can find an extensive discussion in the literature.^8^

The final step is the effective validation of the overall methodology. Here lies another of the main obstacles for the results of fingerprinting to be widely accepted, because validation could be carried out in different ways and using different approaches. This issue will be further discussed in the last section devoted to future perspectives.

### Chromatographic Fingerprinting and Non-targeted Chromatographic Methods

In chromatographic analysis of food, there is a clear parallelism between the underlying meaning of the terms fingerprinting and non-targeted analytical method.^1^ However, in our opinion, the process involved in each is different in scope, and the choice of one or the other should be carefully considered. The adjective “non-targeted” (notice that the terms “non-targeted” and “untargeted” are also used as synonyms), which comes from metabolomics, qualifies an analytical method that is aimed at obtaining technically feasible information on the maximum number of components (originally metabolites) of a material in a single chromatogram.^9^ The output of a non-targeted method could be a fingerprint^10^ provided that the chromatogram is subsequently used in a way consistent with the approach described above, otherwise fingerprinting would not be addressed.^11^ Note that fingerprinting involves applying an overall analytical methodology focused on ensuring the identity of a food product, so that any potential deviation from the identity may be revealed, i.e., adulteration, contamination, mislabeling or misleading labeling claims, etc.

In this sense, chromatography practitioners should keep in mind that the development and optimization of a chromatographic method may be aimed at different targets, depending upon whether the chromatogram is to be used conventionally (targeted analysis) or as a food fingerprint (non-targeted analysis), because the objectives of both may not be mutually compatible. For chromatographic fingerprinting, run time is a crucial variable, even if the chromatographic resolution is sacrificed. Raw chromatograms obtained in the shortest possible time should be pursued, limiting the inclusion of relevant information, although a poor resolution is evident. Ideally, run times of less than 10 min should be aimed at achieving the method competitive with methods based on other analytical techniques, e.g., spectroscopic or spectrometric techniques, although this limit can be increased depending upon the complexity of the material under study. This aspect is not yet clear at present in the minds of many researchers and method developers who continue to perform the same optimization criteria on the belief that the chromatogram obtained can later be used to select chemical markers or identify components that are initially unknown. This objective is valid, and the method is rightly qualified as non-targeted, because it is not driven toward defined analytes, but the main goal of the fingerprinting methodology is divergent. Therefore, it has been rightly proposed to use non-targeted profiling to refer to this particular approach.^12^

In certain cases, the applied method is intended to double check for the presence of known and previously selected analytes and to obtain signals from unknown components. This dual use has been termed as a combined non-targeted and targeted approach.^13^ However, the methods developed in this way usually require long analysis times and, although they provide a characteristic fingerprint, could hardly ever be routinely used.

**Figure 2:**
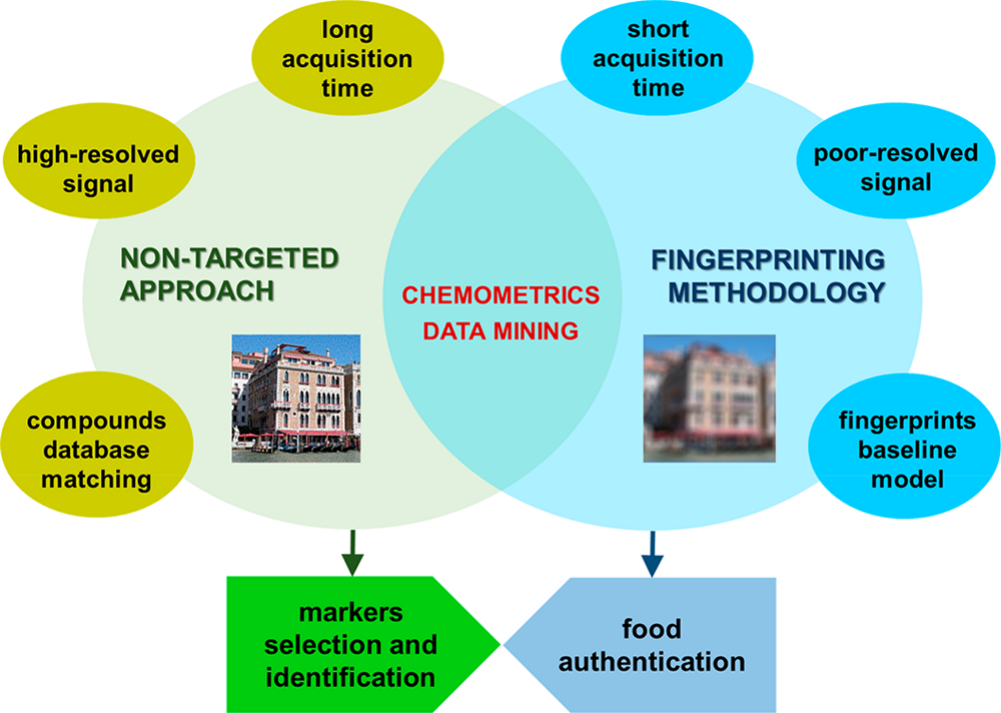
Description of fingerprinting-based and non-targeted chromatographic methods.

Figure 2 graphically illustrates the similarities, differences, and overlaps between fingerprinting and non-targeted chromatographic methods.

### Chemometrics for Fingerprinting

To be mathematically handled, the fingerprint characteristic of each particular food sample is arranged in a mathematical structure generically called a data tensor, which can have different dimensionality depending upon the number of variables defining each signal intensity value. Thus, a 2D fingerprint is disposed in a data vector or first-order tensor, while a 3D fingerprint is defined by a data matrix or second-order tensor. In addition, a set of vectors or matrices obtained from different food samples can be grouped together and then constitute a data array. A more in-depth description is beyond the scope of this perspective but can be easily found in specialized literature.^1,6,7^

**Figure 3:**
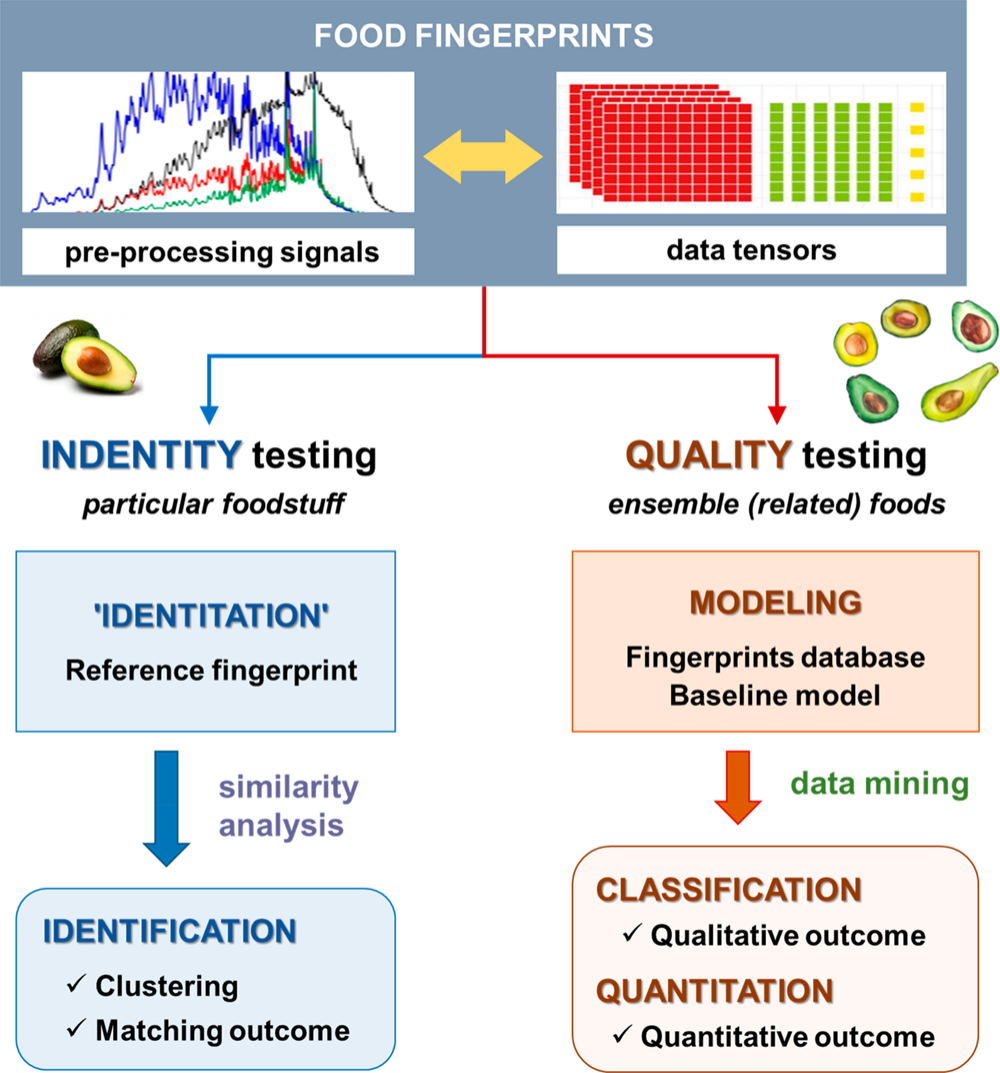
Categories of fingerprinting-based methods aimed at food authentication: identity testing and quality testing.

Once the fingerprints are embedded into data tensors, it is feasible to apply different chemometric method handling for food authentication purposes, which could be gathered into two major categories, identity testing and quality testing, depending upon whether they apply to a particular foodstuff or to a set of foods sharing a common essence. Although both will be briefly discussed below, Figure 3 summarizes the main characteristics of each.

### Identity Testing of Food Products: Identitation and Identification

Identity refers to oneness in all of the traits that comprise the factual singularity of something. Identity is recognized by the set of distinguishing features of a particular material or product. By extension, proving identity relies on verifying sameness with another previously described material or product constituting a reference.

This leads to consideration of two stages to effectively perform food identity testing. The first stage is focused on generating a representative reference of the uniqueness of the product concerned. Notice that, if the final analytical information implies having quantitative values for one or more identity-related features, this reference is based on the definition of certain limiting threshold values. However, when fingerprinting is applied, the aforementioned strategy is not feasible because no specific information is handled and the reference is then likened to a given fingerprint. The second stage requires verifying the consistency between the fingerprint obtained from the food under testing and the reference fingerprint.

To distinguish between the two stages, the terms “identitation” and “identification” were proposed. “Identitation” refers to defining the identity of a material or product on the basis of measurable features, i.e., a chromatographic fingerprint.^6,14^ Complementarily, “identification” involves a comparison of such features, i.e., carrying out a similarity analysis between pre-processed chromatograms (fingerprints), which results in the computation of proper similarity indices or matching indices, usually normalized to the 0–1 range. Performing cluster analysis is also advisible to have a quick screening. It is not possible, therefore, to perform “identification” without having previously carried out “identitation”.

### Quality Testing of Ensemble Foods: Data Mining

Food authentication does not usually involve a single product but is applied to sets of foods sharing one or more features, e.g., botanical, animal, or geographical origins, ingredients, quality-differentiated indications, health claims, manufacturing, etc. When this happens, application of data-mining methods is required. They are able to carry out a mapping of the shared patterns in the fingerprint, locate the information that is common in the ensemble foods, differentiate it from those that do not comply with this requirement, and finally extract it. This process as a whole constitutes the quality testing, and multivariate data processing tools, generically called pattern recognition, are applied. All of them are based on the application of artificial intelligence methods, currently so-called machine-learning methods.

The effective implementation of quality testing includes three steps. The first step involves creating a database of diverse but representative fingerprints of the ensemble, measured on food commodities that have previously been stated to be authentic or genuine (i.e., they gather the characteristics that determine the common essence of the ensemble foods). This step is critical because it becomes the reference against which subsequent decisions will be based.

The second step is typical of the quality testing and involves building a multivariate model that fits the information on interest included in the fingerprint database. This is in essence a statistical regression-based process and can give rise to qualitative models (classification models) or quantitative models (quantitation models).^2^ Note that it is usual in the literature to link regression only with quantitative models, but in our opinion, it may be applied to both types of models because there is no conceptual difference. The same database enables the building of as many food-specific models, also so-called baseline fingerprints, as features to be modeled. The second stage is further decomposed into two sub-steps, which are called training (also so-called calibration, particularly in the case of quantitative models) and validation (or testing). For this purpose, the database is split into two equivalent sub-sets regarding the representativeness of the feature to be modeled, which are called the training set and the validation set.

In the third step, the model is applied on food samples, consistent with the modeled food commodities, to assign or predict the feature considered (quality or quantity, respectively), which constitutes the outcome of the quality testing.

There is currently an extensive battery of multivariate methods that can be applied. Among them that could be highlighted are those that have traditionally been applied in the field of conventional chemometrics,^15,16^ e.g., *k*-nearest neighbor (KNN), linear or quadratic discriminant analysis (LDA or QDA), soft independent modeling of class analogy (SIMCA), or partial least squares discriminant analysis (PLS-DA), to mention some of the most used for classification models, or partial least squares regression (PLSR) for quantitation models. In addition, a number of alternative methods have recently been included,^17^ such as support vector machine (SVM), classification and regression tree (CART), random forest (RT), or artificial neural network (ANN), which are used for both qualitative and quantitative models. The choice of one or another multivariate method depends upon many factors, among them, especially the number and representativeness of the data available to train the model. It is generally advisible to try more than one, because it is difficult to know *a priori* which one will perform better in each specific case.

A recent advance is given by the ability of combining fingerprints obtained from different chromatographic systems to compose a supra-fingerprint. For this purpose, data fusion methods are applied, within which three levels are defined depending upon whether the original fingerprints are used for the fusion model (low level) or the previously extracted information (medium and high levels).^18^ Detailed information on the peculiarities of each of the methods mentioned in this section is again beyond the scope of this perspective and should be consulted in the specialized literature that has been referenced.

### State of the Art of Food Chromatographic Fingerprinting

As already mentioned, fingerprinting was born in the metabolomics field at the end of the 20th century but was soon incorporated into the vocabulary of food authentication or food forensics. However, it was not until about a decade ago that the first specific and metabolomic-independent review was published,^19^ although only vibrational spectroscopy [infrared (IR) and Raman], NMR, and MS were considered as suitable analytical techniques. The first comprehensive review on the use of chromatographic fingerprinting in food authentication was possibly released in 2016,^6^ and since then, to the authors’ knowledge, only a few reviews with a more restricted scope have been reported, i.e., dedicated to conventional liquid chromatography (LC),^20^ gas chromatography (GC),^21^ and comprehensive two-dimensional gas chromatography (2D GC).^1^ These reviews mostly gather studies that are applying quality testing, and a few studies have been reported addressing identity testing. Some of the few examples available concern the verification of homogeneity and stability of olive oil reference materials.^22,23^

There are as of yet few studies addressing chromatographic fingerprint fusion, despite its high potential. In fact, only three articles dealing with the authentication of edible vegetable oils and coffee have been found. In the first article, LC fingerprints measured with two complementary detectors, diode array ultraviolet (UV) absorption (DAD) and charged aerosol (CAD), are fused to discriminate the geographical origin (Asia, Africa, and America) of palm oils.^24^ In the second article, GC fingerprints measured with a flame ionization detector (FID) are fused with LC–CAD fingerprints to differentiate olive oils of the same botanical variety (Arbequina) harvested in two non-adjacent geographical regions from Spain.^25^ Finally, the third paper merges two chromatograms of volatile and non-volatile compounds, obtained using headspace/solid-phase microextraction–gas chromatography–mass spectrometry (HS/SPME–GC–MS) and liquid chromatography–diode array UV absorption (LC–DAD), respectively, to correlate chemical data with odor and taste attributes.^26^

## Future Perspectives: Transfer and Implementation

Despite the major progress achieved in the development of food fingerprinting, the transfer from academia to the real analytical world, mainly to forensic food laboratories performing routine quality, authenticity, and safety food control, is still pending.^5^ The key reason may be the lack of confidence that still remains on the reliability of those scientific–technical outcomes not based on clearly and evidently perceived information. This mistrust is more surprising among many scientists and analytical chemistry practitioners, which increases the difficulty for such a methodology to be accepted. This has led to consideration of non-targeted methods based on chromatographic fingerprints only as a first step for chemical marker identification that could later be used in targeted methods.^5^

A direct consequence of this situation is the almost generalized absence of fingerprinting in the catalog of official analytical methods for food control. This topic was already addressed in 2014 in an excellent tutorial,^27^ and surprisingly, the situation has hardly changed since then. An additional drawback is the difficulty of mainstreaming these analytical methods into good laboratory practice (GLP) or ISO 17025 laboratory accreditation schemas. All of this means that the analytical results have no legal recognition in technical or commercial disputes nor in legal litigations, which prevents their effective implementation and forensic use.^3^

However, the recent outbreak of artificial intelligence in all fields of science and technology may rethink the need for fingerprinting methodology as a valid tool for food forensic laboratories. Beyond other considerations, this opens the door to the incorporation in laboratories of staff members with expertise in processing and storing large amounts of analytical data and who should not be outsiders to analytical practice.

From a practical point of view, the transferability and implementation of fingerprinting-based analytical methods relies on two cornerstones still to be developed: harmonization (including validation) and databases, which will be concisely discussed below.

### Harmonization of Fingerprinting-Based Chromatographic Analytical Methods

Analytical harmonization involves an effort to establish rules and requirements to ensure that analytical methods are the same or similar or eventually consistent when applied in different laboratories or entities, so that the results are comparable.

**Figure 4:**
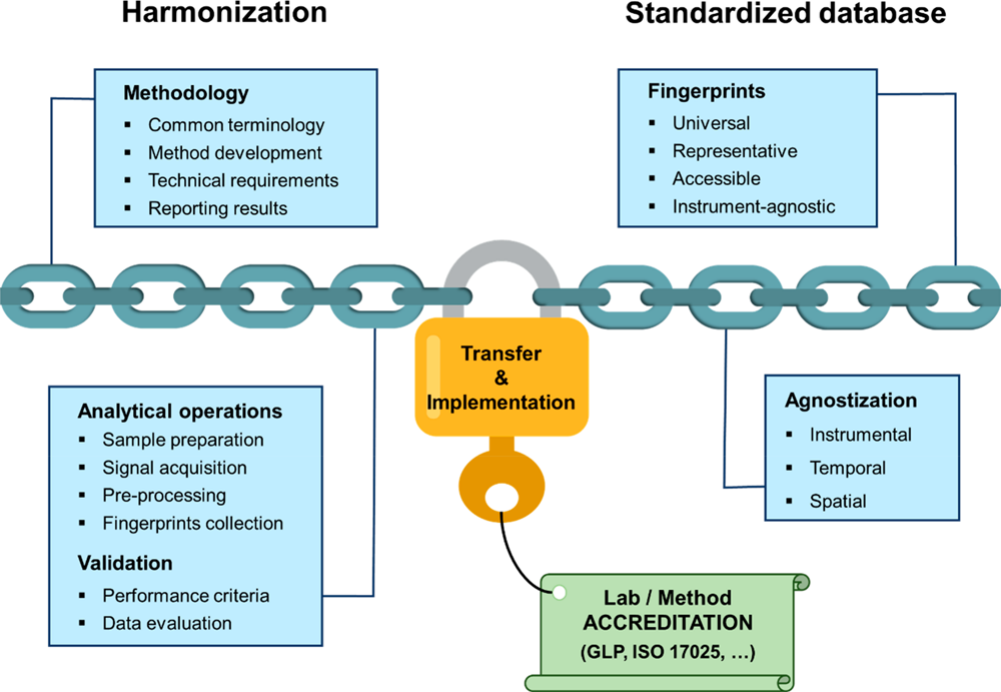
Elements to be considered for harmonizing food fingerprinting approaches aimed at implementation in forensic food laboratories.

In the context of food fingerprinting approaches, harmonization involves having standardized protocols that (i) establish a common terminology, (ii) provide guidance for the development of such methods, (iii) specify requirements, and (iv) define how results should be reported. This would lead to fingerprinting-based methods being recognized and accepted. Figure 4 shows graphically the different elements involved in the harmonization of fingerprinting.

In this regard, the *Food Chemicals Codex (FCC)*, under the United States Pharmacopeia (USP), maintained since 2017 a recent guidance on developing and validating non-targeted methods^28^ that could be considered as the first guideline dealing with harmonization, although it is aimed only at methods for detecting food adulteration. On the basis of this directive, AOAC International has published since 2020 four standard method performance requirements for non-targeted testing of ingredients for food authenticity/fraud evaluation of honey, extra virgin olive oil, pasteurized whole liquid bovine milk, and vanilla powder and extracts (https://www.aoac.org/resources/).

A crucial issue of harmonization is the validation of the methodology candidate to be implemented. This topic has been discussed in some tutorials^29,30^ and is specifically addressed in the *FCC* guidance.^28^ Because of the wide scope of purposes of fingerprinting methods, it is not possible to consider a single validation approach but rather, depending upon the intended application, the validation procedure to be applied differs. It should be reminded that there are two types of testing: identity testing and quality testing, and that either of them could be qualitative or quantitative. In addition, this challenge is increased if the distinctive nature of chromatographic methods is also taken into account.

To elaborate on the subject, a distinction between the (statistical) validation of multivariate models^31^ and the (analytical) validation of results can be performed. Leaving aside the first one, the analytical validation of quantitative results could be adapted with low effort from the guidelines for the targeted methods. However, the same is not the case for qualitative methods that require specific consideration and for which the traditional concepts of traceability and uncertainty are diluted. In practice, uncertainty cannot be applied, and instead, the notion of certainty should be incorporated. In this way, a representative parameter based on the probability of obtaining correct results, i.e., the belonging of a food sample to a certain class, should be defined to support any qualitative outcome.

A recent validation approach that distinguishes two scenarios for qualitative methods has recently been published.^32^ These scenarios reflect both producer and user interests and focus on facets such as conformity assessment and marketing cost-effectiveness. For each of these scenarios, quality indicators of the analytical results have been defined, and from them, some fitness-for-purpose criteria of analytical method performance are set. This proposal has clear practical implications and is a valuable attempt to rationalize the validation of qualitative methods for implementation and acceptance goals.

Two studies aimed at the harmonization of non-targeted methods for food authentication based on MS^33^ and NMR^34^ analyses have already been reported, which is undoubtedly excellent news. However, any proposal focused on the use of chromatographic fingerprinting would be welcome at this time because, as far as the authors are aware, none has yet been published, and further research in this regard should be a priority matter.

### Standardized Databases of Chromatographic Fingerprints

The reliability of the results found from the right interpretation of fingerprinting-based methods relies on the availability of universal, recognized, and accessible databases collecting a sufficient number of fingerprints representative of the food or the ensemble foods concerned. However, the only precedent of fingerprint databases covers chromatograms obtained by thin-layer chromatography (TLC) and high-performance liquid chromatography (HPLC) and is intended for the botanical identification of medicinal plants.^35^

However, to date there are no similar databases from specific food fingerprints, and each laboratory or entity generates and uses its own database. In principle, such databases would be easier to create from spectroscopic or spectrometric signals that are stable and highly reproducible once certain instrumental conditions are set. However, with regard to chromatographic fingerprints, the task presents numerous additional problems because chromatograms are dependent upon each instrument type and the state of the column and detector. Indeed, chromatograms of the same food sample obtained in different laboratories or instruments or in the same instrument but in a large time interval are not the same and show deviations in both retention times and signal intensities. As a result, chromatographic signals should be previously standardized, so that they may be included in an easy to access database.

To date, there are not many approaches describing how such standardization should be carried out. One of the latest published suggests performing a double standardization in times and intensities, using easily accessible chemical references that are ad-hoc-defined. In this way, instrument-independent fingerprints are achieved that have been termed instrument-agnostic fingerprints.^36,37^
